# Unraveling Metabolic Dysfunction-Associated Steatotic Liver Disease Through the Use of Omics Technologies

**DOI:** 10.3390/ijms26041589

**Published:** 2025-02-13

**Authors:** Maria V. Bourganou, Maria Eleni Chondrogianni, Ioannis Kyrou, Christina-Maria Flessa, Antonios Chatzigeorgiou, Evangelos Oikonomou, Vaia Lambadiari, Harpal S. Randeva, Eva Kassi

**Affiliations:** 1 Department of Biological Chemistry, Medical School, National and Kapodistrian University of Athens, 11527 Athens, Greece; mbourganou@med.uoa.gr (M.V.B.); marielena.hondr@gmail.com (M.E.C.); cmflessa@gmail.com (C.-M.F.); 2 Endocrine Unit, 1st Department of Propaedeutic Internal Medicine, Laiko Hospital, National and Kapodistrian University of Athens, 11527 Athens, Greece; 3 Laboratory of Dietetics and Quality of Life, Department of Food Science and Human Nutrition, School of Food and Nutritional Sciences, Agricultural University of Athens, 11855 Athens, Greece; 4 Warwickshire Institute for the Study of Diabetes, Endocrinology and Metabolism (WISDEM), University Hospitals Coventry and Warwickshire NHS Trust, Coventry CV2 2DX, UK; 5 Institute for Cardiometabolic Medicine, University Hospitals Coventry and Warwickshire NHS Trust, Coventry CV2 2DX, UK; 6 Warwick Medical School, University of Warwick, Coventry CV4 7AL, UK; 7 Centre for Health & Life Sciences, Coventry University, Coventry CV1 5FB, UK; 8 Aston Medical School, College of Health and Life Sciences, Aston University, Birmingham B4 7ET, UK; 9 College of Health, Psychology and Social Care, University of Derby, Derby DE22 IGB, UK; 10 Department of Physiology, Medical School, National and Kapodistrian University of Athens, 75 Mikras Asias Str., 11527 Athens, Greece; achatzig@med.uoa.gr; 11 3rd Department of Cardiology, “Sotiria” Thoracic Diseases Hospital of Athens, Medical School, National and Kapodistrian University of Athens, 11527 Athens, Greece; boikono@gmail.com; 12 2nd Department of Internal-Medicine, Diabetes Centre, Attikon University Hospital, Medical School, National and Kapodistrian University of Athens, 12462 Athens, Greece; vlambadiar@med.uoa.gr

**Keywords:** metabolic dysfunction-associated fatty liver disease (MAFLD), metabolic dysfunction-associated steatotic liver disease (MASLD), non-alcoholic fatty liver disease (NAFLD), omics technologies, genomics, transcriptomics, proteomics, metabolomics, exposomics, biomarkers

## Abstract

Non-alcoholic fatty liver disease (NAFLD), now referred to as metabolic dysfunction-associated steatotic liver disease (MASLD), is the most prevalent liver disorder globally, linked to obesity, type 2 diabetes, and cardiovascular risk. Understanding its potential progression from simple steatosis to cirrhosis and hepatocellular carcinoma (HCC) is crucial for patient management and treatment strategies. The disease’s complexity requires innovative approaches for early detection and personalized care. Omics technologies—such as genomics, transcriptomics, proteomics, metabolomics, and exposomics—are revolutionizing the study of MASLD. These high-throughput techniques allow for a deeper exploration of the molecular mechanisms driving disease progression. Genomics can identify genetic predispositions, whilst transcriptomics and proteomics reveal changes in gene expression and protein profiles during disease evolution. Metabolomics offers insights into the metabolic alterations associated with MASLD, while exposomics links environmental exposures to MASLD progression and pathology. By integrating data from various omics platforms, researchers can map out the intricate biochemical pathways involved in liver disease progression. This review discusses the roles of omics technologies in enhancing the understanding of disease progression and highlights potential diagnostic and therapeutic targets within the MASLD spectrum, emphasizing the need for non-invasive tools in disease staging and treatment development.

## 1. Introduction

### 1.1. The Evolution of Liver Disease Nomenclature: From NAFLD to MASLD

Non-alcoholic fatty liver disease (NAFLD) is considered the most common liver disease worldwide and the leading cause of liver-associated morbidity and mortality [1,2,3]. One-third of the global population is considered to be affected by NAFLD [1,2]. The prevalence of NAFLD increases in parallel with those of obesity, type 2 diabetes, dyslipidemia, and hypertension and often occurs in combination with at least one of these conditions [4,5,6]. Notably, the incidence of NAFLD among adults with at least one of these cardio-metabolic conditions rises significantly to over 60–75% [7,8].

**Figure 1:**
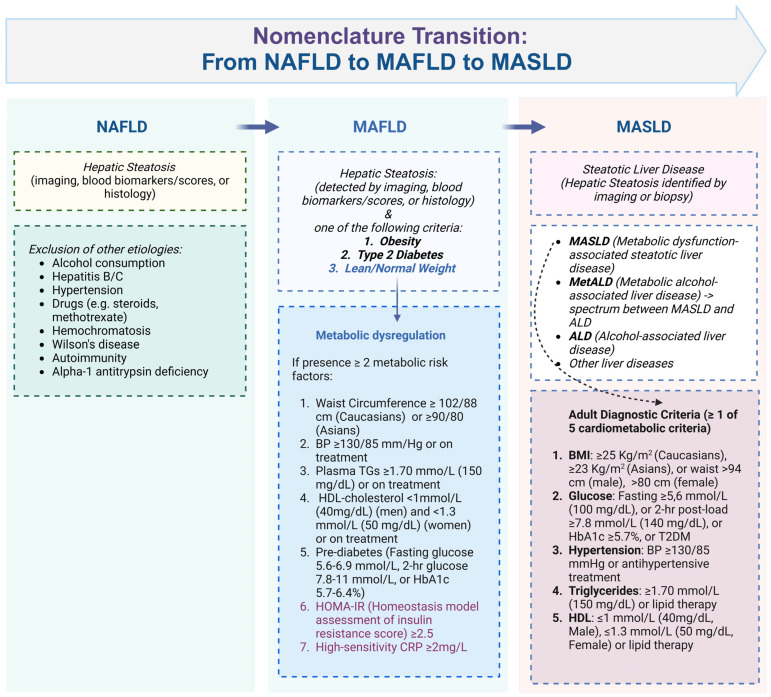
Nomenclature transition: from NAFLD to MAFLD to MASLD. Created in BioRender. https://BioRender.com/q37n868 (accessed on 11 February 2025).

Since the inception of the term NAFLD, extensive discussions have ensued regarding the nomenclature of fatty liver disease (FLD). In 2020, a consortium of international experts released a statement proposing a change from NAFLD to metabolic dysfunction-associated fatty liver disease (MAFLD) to better reflect its association with cardio-metabolic diseases and risk factors. These risk factors include increased body mass index (BMI) or waist circumference, hypertension, high plasma triglycerides, low HDL cholesterol, prediabetes, and insulin resistance [9]. The diagnostic criteria for MAFLD require evidence of hepatic steatosis—confirmed through histology, imaging, or biomarkers—alongside one of the following conditions: overweight/obesity, type 2 diabetes mellitus (T2DM), or metabolic dysregulation. Metabolic dysregulation is defined by the presence of at least two metabolic risk factors, as outlined in Figure 1 [9].

This proposal aimed to highlight the diverse nature of NAFLD and its link to metabolic risk factors that may coincide with other liver conditions, like alcohol-related liver disease (ALD).

While the adoption of the MAFLD term has been somewhat widespread, concerns emerged about the implication of different causes and the use of the term “fatty” in the revised nomenclature, as it was seen as stigmatizing. To address these concerns, a collaborative Delphi statement by multiple societies was released in 2023, suggesting a further change of the nomenclature to metabolic dysfunction-associated steatotic liver disease (MASLD) [8].

Subsequently, the term MASLD, which erases the term “fatty”, has been adopted with diagnostic criteria requiring the presence of hepatic steatosis and at least one of five cardiovascular risk factors, which include BMI ≥ 25 kg/m^2^ for Caucasians (≥23 kg/m^2^ for Asians) or waist circumference >94 cm in men and >80 cm in women, elevated fasting glucose or HbA1c, T2DM, hypertension (≥130/85 mmHg or treated), high triglycerides (≥1.70 mmol/L or treated), and low HDL cholesterol (≤1.0 mmol/L in men or ≤1.3 mmol/L in women or treated) [10]. Additionally, patients fulfilling both MASLD and alcohol-associated liver disease (ALD) criteria are classified under the category of MetALD [11].

In the wake of these recent MASLD nomenclature changes, there is a growing but still limited body of research examining MASLD in comparison to NAFLD or MAFLD in order to address questions about whether the evidence gathered under the NAFLD nomenclature can be directly applied to MASLD [12].

### 1.2. Prevalence, Race, and Ethnicity of the Disease

The global prevalence of NAFLD is estimated at 32%, with higher rates in males than females. The prevalence has increased over time and is projected to continue rising if current trends persist [13,14].

The etiology of NAFLD is multifaceted, influenced by both genetic and environmental factors that affect its incidence and progression [15,16]. Regarding the prevalence and severity of NAFLD, numerous studies indicate racial or ethnic variations potentially attributable to lifestyle, dietary habits, metabolic comorbidities, and genetics [14]. Research on these disparities provides insight into biological and genetic differences among individuals with similar cultural, dietary, and geographic backgrounds [14]. However, most studies are limited to specific countries, and findings may not be universally applicable due to environmental, socioeconomic, and healthcare policy differences [14].

The comparative epidemiologic research on NAFLD prevalence across ethnicities is limited and is predominantly within the United States [14]. Race/ethnicity research is critical in heterogeneous nations like the U.S., as it elucidates biological, pathophysiological, and risk factor variations among ethnic groups [14,17]. This knowledge enhances the understanding of disease etiology and the interaction of genetic and environmental influences, thus facilitating the application of global data for public health initiatives aimed at prevention, screening, and treatment programs [14,17].

A meta-analysis conducted by Rich et al. (2018) found significant racial and ethnic disparities in the prevalence and severity of NAFLD in the U.S., with the highest prevalence observed in Hispanic individuals (22.9%), followed by those of European origin (14.4%) and African origin (13.0%). Among NAFLD patients, Hispanics faced a slightly higher risk of NASH (RR: 1.09), whereas Africans had a lower risk (RR: 0.72) compared to Caucasians. However, significant fibrosis rates showed no clear racial or ethnic differences, and available data on NAFLD outcomes remained limited and inconsistent [18].

In alignment with the prior study, data from the National Health and Nutrition Examination Surveys (NHANES) 2017–2018 showed that NAFLD prevalence was highest among Hispanics (63.7%), followed by individuals of European descent (56.8%) and those of African descent (46.2%), influenced by genetic and metabolic factors [19]. This may be due to genetic factors, such as the PNPLA3 mutation, which is linked to an increased risk of hepatic steatosis and NASH, particularly in Hispanics [20,21]. Metabolic factors, including increased central adiposity and insulin resistance, are also more prevalent in Hispanics than in Caucasians [22,23]. Additionally, lower serum triglyceride levels in African Americans may lead to a decreased prevalence of NAFLD [24].

The prevalence of NAFLD in South America is estimated to be as high as 59%, according to a meta-analysis, though this figure may be influenced by hospital-based studies focusing on high-risk individuals [25]. Le et al. estimated a lower but still significant prevalence of 35.7%, likely driven by genetic susceptibility, high rates of obesity, metabolic risk factors, and physical inactivity [13,26,27,28]. The PNPLA3 genetic polymorphism is highly prevalent in the general population, particularly in those of Native American descent [29].

The prevalence of NAFLD in Europe ranges from 26.9% to 32.6% based on meta-analyses [2,26,30]. Turkey exhibited the highest prevalence of NAFLD at 48.4%, with Italy following at 38.2% and France presenting a lower prevalence of 18.2% [2,13,31].

The prevalence of NAFLD in Asia is approximately 30%, with significant variability across countries [14,26,32]. Iran has the highest prevalence at around 38.07%, while Japan has the lowest at 22.3% [32].

The global prevalence of NAFLD is increasing globally despite regional differences [26]. Comprehensive strategies are essential to combat this trend, which involve raising awareness among healthcare practitioners and the public, improving non-invasive diagnostic methods, and identifying effective treatments for NAFLD [26].

### 1.3. The Landscape of MASLD: Pathogenesis and Diagnosis

NAFLD includes a wide spectrum of diseases, ranging from simple steatosis to steatohepatitis and cirrhosis, which can even progress to hepatocellular carcinoma (HCC).

NAFLD is distinguished by the excessive accumulation of fat in the liver, coupled with insulin resistance and the existence of steatosis in more than 5% of hepatocytes. Within the realm of NAFLD, there are two distinct pathological states with differing prognoses: non-alcoholic fatty liver (NAFL) and non-alcoholic steatohepatitis (NASH), the latter encompassing a wide range of disease severity, such as fibrosis, cirrhosis, and even related hepatocellular carcinoma (HCC) [33]. NASH is defined by the presence of hepatic steatosis (more than 5%) combined with inflammation and hepatocyte injury (ballooning), with or without fibrosis [34]. Cirrhosis is a progressive stage of NASH with fibrosis of the liver due to long-term tissue damage. Similarly, the disease spectrum of MAFLD and MASLD signifies a continuum ranging from hepatic steatosis to metabolic-associated steatohepatitis (MASH) with potential progression to fibrosis, cirrhosis, and HCC. The comprehension of the progressive nature of NAFLD/MAFLD/MASLD is crucial for effective patient care, driving the development of diagnostic tools for various underlying pathologies like steatosis, steatohepatitis, and fibrosis. Of note, the assumption of a linear progression in NAFLD/MAFLD/MASLD is challenged, as evidence suggests a more complex reality with potential progression, regression, or stability. Thus, further research is necessary to assess the variability in the efficacy of non-invasive serum biomarkers and scoring systems across the diagnostic classifications of NAFLD, MAFLD, and MASLD [10].

NASH constitutes the most rapidly growing cause of liver disease that necessitates liver transplantation in the United States [35]. NASH is strongly connected with metabolic syndrome and all the relevant co-morbidities, such as obesity, type 2 diabetes, dyslipidemia, and cardiovascular disease; therefore, NASH patients are at high risk of morbidity and mortality. In addition, NASH patients were associated with an increased risk of developing post-liver transplantation malignancy. Based on a large cohort study, ethnicity, race, multiple organ transplantation, and previous history of malignancy also play a crucial role in cancer risk after liver transplantation [36].

The pathophysiological mechanisms linking these conditions to adverse outcomes remain incompletely understood, although inflammation and worsening insulin resistance play significant roles. It is of particular interest that accurate and prompt diagnosis plays a critical role in managing hepatic complications and reducing the compounded risk of cardiovascular disease linked to NAFLD/MAFLD/MASLD and metabolic syndrome [10].

The pathogenesis of NAFLD is a complex process involving various cardiovascular disease risk factors, such as high-fat diets and low physical activity levels, as well as genetic variations. An imbalance in lipid and glucose metabolism is believed to be at the core of NAFLD development. Metabolic syndrome, particularly type 2 diabetes, impacts glucose and lipid metabolism, as well as the gut microbiota, which appears to contribute to NAFLD pathogenesis through the gut–liver axis. Insulin resistance, prevalent in many individuals with abnormal glucose and lipid metabolism, promotes the conversion of liver free fatty acids to triglycerides intracellularly [5]. Furthermore, insulin resistance is also linked to dysregulated levels of adipokines, including adiponectin, leptin, tumor necrosis factor alpha (TNF-a), interleukin-1β, and interleukin-6, which have been linked to inflammation and fat accumulation [37]. Also noteworthy are additional contributing pathogenetic mechanisms relating to endoplasmic reticulum (ER) stress, apoptosis, and autophagy [38,39,40]. Indeed, disruptions in glucose metabolism progressively cause increasing ER stress, inflammation, and heightened oxidative stress.

Despite its potential progression and complications, NAFLD often remains asymptomatic for several years or even decades; thus, it is often overlooked with marked delays in necessary management/lifestyle changes [37]. The diagnosis of NAFLD relies on radiological/imaging methods like liver ultrasound, computed tomography, or magnetic resonance imaging and can be histologically confirmed by liver biopsy. Moreover, the diagnosis of NAFLD requires the exclusion of excessive alcohol consumption, drug-related hepatotoxicity, viral hepatitis, and autoimmune or other similar conditions that could cause liver disease. However, the identification of MAFLD and MASLD recognizes the multifactorial nature of hepatic injury in various cases, where metabolic abnormalities and alcohol-related factors both play a role. It is noteworthy that this classification is rooted in the presence of metabolic dysfunction rather than the absence of alternative liver pathologies [41,42].

The severity of hepatic necrotizing inflammation and fibrosis significantly impacts the long-term outlook for patients with NAFLD. Consequently, timely monitoring of disease progression and intervention are crucial. While liver biopsy has traditionally served as the gold standard for NAFLD diagnosis, its widespread clinical implementation remains challenging primarily due to its invasive nature. However, advancements in omics-related research technologies have unveiled the potential diagnostic value of omics biomarkers, including genomics, transcriptomics, proteomics, and metabolomics, in NAFLD diagnosis [43].

Notably, NAFLD and NASH are intricate disorders characterized by modifications in the expression of numerous genes and an array of proteins encoded by these genes. Prior to the era of high-throughput analysis, conventional translational research efforts aimed at identifying molecular targets for such intricate diseases were carried out in a gene-specific manner. However, with the introduction of innovative technologies like expression microarrays, mass spectrometry, and reverse proteomics, researchers can now uncover intricate patterns in the manifestation of biologically active molecules. As a result, high-throughput approaches are highly appropriate for investigations aimed at revealing the molecular foundations of persistent liver conditions like NAFLD [44].

### 1.4. Omics Technologies

**Figure 2:**
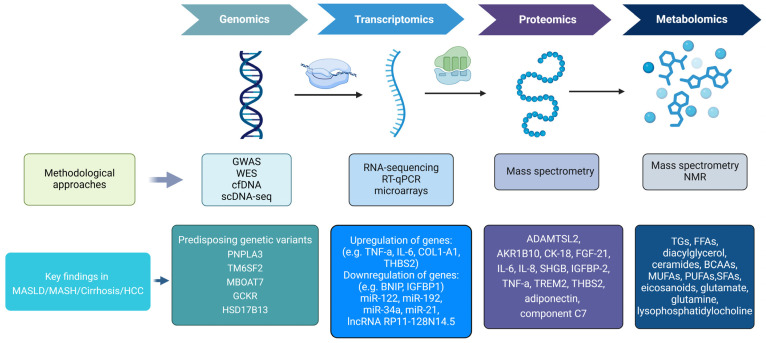
Advancements in Omics Technologies for MASLD Research: Methodologies and Key Findings in MASLD, MASH, Cirrhosis and HCC. Created in BioRender, https://BioRender.com/y12y360 (accessed on 12 February 2025).

Omics technologies are high-throughput technologies that have been applied for the identification of biomarkers, the characterization of biochemical reactions, and the clarification of many pathophysiological processes [45]. These non-invasive diagnostic modalities offer a new tool to better comprehend the distinct stages of NAFLD and can be used for the discovery of new treatments. The most common omics technologies applied in NAFLD research include genomics, transcriptomics, proteomics, and metabolomics. These techniques can contribute to better understanding, diagnosing, and treating NAFLD. Indeed, omics technologies are expected to bring us a step closer to better understanding the pathophysiology of NAFLD and further discovering non-invasive methods for the diagnosis of the disease, the recognition of its different stages, and the identification of therapeutic targets [46]. For example, various omics technologies applied in in vitro NAFLD models provide further insights into the underlying mechanisms of NAFLD, which may facilitate the discovery of biomarkers for the diagnosis of the disease, as well as the development of novel drugs against NAFLD [47]. Figure 2 depicts the main methodologies, and key findings in MASLD, MASH, cirrhosis, and HCC.

The application of omics technologies markedly improves the understanding and oversight of MASLD, while such advancements pave the way for more accurate diagnostics, targeted therapies, and, ultimately, better outcomes for patients. This review outlines the key discoveries from omics technologies relevant to MASLD, with a particular focus on genomics, transcriptomics, proteomics, and metabolomics (lipidomics and glycomics) and the role of exposomics in the field of MASLD.

## 2. Genomics and MASLD

Genomics is a foundational and extensively researched omics discipline and has provided significant insights into the link between specific single nucleotide polymorphisms (SNPs) and NAFLD development [46]. In the era of personalized treatment, genomics will soon be employed to identify people who are at heightened risk for a more aggressive progression of NAFLD and in the prediction of responses to specific treatments [46]. This approach may require enhanced monitoring and inform treatment choices for high-risk cohorts [46].

The genetics of NAFLD is of significant interest in its pathophysiological assessment. Numerous genome-wide association and candidate gene studies have identified SNPs associated with NAFLD’s onset, severity, and clinical manifestations [48]. Additionally, numerous liver disease-associated gene variants have been discovered through exome-wide association studies, linking gene variants to patient [49,50].

Genomic studies have elucidated cellular responses to excessive hepatic fat deposition in NAFLD and revealed gene regulation associated with lipid droplet formation and NAFLD-related alterations in mitochondrial lipid and glucose metabolism [51]. In addition, genomic studies have identified numerous therapeutic drug targets. Additionally, recent studies aimed to develop polygenic risk scores that integrate clinical parameters to improve predictive accuracy. However, challenges remain in interpreting these genetic scores, their use in risk stratification as opposed to predictive modeling, and their restricted applicability primarily due to a predominance of studies conducted on European populations [52].

GWAS have demonstrated robust links between NAFLD progression and variations in certain genes, including PNPLA3, TM6SF2, MBOAT7, GCKR, and HSD17B13 [53].

### 2.1. PNPLA3

Notably, the PNPLA3 (Palatin-like phospholipase domain-containing 3) gene is the predominant genetic factor influencing the onset and progression of NAFLD to NASH fibrosis and potentially HCC [54]. Positioned in the 22q13 section of chromosome 22, it encodes adiponutrin (ADPN), which exhibits heightened lysophosphatidic acid acyltransferase activity (LPAAT), resulting in an increase in intracellular lipid accumulation. Within the *PNPLA3* gene, it exists in a non-synonymous SNP (rs738409 C/G), a coding variant that encodes an amino acid substitution I148M and shows a strong correlation with fatty liver disease and its histological severity in both adults and children [55]. The I148M substitution is known to enhance hepatic lipid synthesis by means of a gain in function, providing a plausible biochemical explanation for the development of hepatic steatosis in those carrying the I148M variant (rs738409, allele G) [56]. The adiponutrin variant p.I148M (rs738409) alters the composition of hepatic lipids by reducing the transfer of polyunsaturated fatty acids (PUFAs) from diacylglycerols (DAGs) to phosphatidylcholine (PC). Consequently, this leads to the increased PUFA content of triglycerides (TGs) and DAGs, alongside a disruption in phosphatidylcholine synthesis and the inhibition of lipid droplet hydrolysis [57].

Based on a large ethnically diverse cohort study, the I148M risk allele was most prevalent in Hispanics, intermediate in Caucasians, and lowest in African Americans [58]. Recently, GWAS data indicated a predominance in the allele among Hispanic patients [59].

As referenced above, one of the major and primary genetic determinants contributing to NAFLD is SNP (rs738409) in the *PNPLA3* gene. More specifically, the PNPLA3 rs738409 C > G SNP is classified as a missense mutation, leading to the substitution of cytosine with guanosine. Consequently, this substitution causes the false encoding of methionine instead of isoleucine at position 148. Concerning the genotype, though, a meta-analysis of people carrying CG and GG genotypes within a population exhibited an 88% increased likelihood of NAFLD, whereas individuals carrying the CC genotype had a low possibility of developing NAFLD [60]. The implications of the rs738409 G-allele reveal that there are clear correlations between Gamma-glutamyl Transferase (GGT) and transaminases (alanine aminotransferase and serum aspartate aminotransferase), revealing a pro-inflammatory response [20]. A large cohort study conducted in patients diagnosed with NAFLD after histological examination revealed a robust connection between the rs738409 G allele and the presence of steatosis, as well as its correlation with histological severity. The G allele rs738409 can predispose individuals to hepatic fat accumulation, although environmental conditions and other hereditary traits might also have a crucial role in triggering inflammation, cellular damage, or fibrosis. In cases where patients develop NASH, the presence of the rs738409 G allele tends to exacerbate the severity of the disease. Notably, in pediatric patients, the presence of the rs738409 G allele correlates with an earlier onset of the disease [61]. Additionally, the *PNPLA3* rs738409 C > G polymorphism is linked to increased HCC risk in MASLD patients. Conversely, the *PNPLA3* genotype does not affect extrahepatic cancer incidence in MASLD patients [62]. Other variants of the *PNPLA3* gene, including rs2896019, rs381062, rs738408, and rs3747207, appear to be less significant in NAFLD pathogenesis [63,64].

Additionally, genetic models have been established to mimic polymorphisms linked to the development of NAFLD, such as those utilizing mice with the *Pnpla3* polymorphism and leptin- or leptin receptor-deficient mice [65]. These models are instrumental for exploring distinct molecular pathways and elucidating mechanisms that disrupt the hepatic equilibrium and lead to NAFLD progression, as well as understanding the impact of their dysregulation. However, the required genetic manipulation(s) in these models may not fully reflect NAFLD pathophysiology in humans [66]. More specifically, the etiology of human NAFLD is influenced by environmental and genetic factors, with humans typically exhibiting complex conditions that involve multiple pathogenic pathways, including obesity and other risk factors. By contrast, many genetic murine models are monogenic, capturing specific genetic aspects of NAFLD. However, significant gaps remain in understanding how these simplified models reflect the broader complexity observed in human disease [67].

Flessa et al. presented an overview of the most prevalent animal models utilized in NAFLD research, discussing the advantages and disadvantages of each model, as well as the challenges encountered by researchers engaged in the development and utilization of animal models for translational NAFLD research [65]. Regarding advantages, the *Pnpla3* I148M mice characterized by *Pnpla3* I148M overexpression in the liver develop hepatic steatosis with increased levels of triacylglycerol and other lipids. Additionally, *Pnpla3* I148M mice subjected to a high-sucrose diet exhibit even higher levels of triacylglycerol and fatty acids, along with more pronounced steatosis. Conversely, under a high-fat diet, hepatic fat levels in *Pnpla3* I148M mice remained unchanged, and steatosis was absent. These findings suggest that *Pnpla3* I148M mice develop diet-induced hepatic steatosis, highlighting the pivotal role of diet in PNPLA3-polymorphism-related steatosis and underscoring the limitations of these mice as a model that fails to encompass the entire spectrum of NAFLD [65,68,69].

### 2.2. TM6SF2

Transmembrane 6 superfamily member 2 (*TM6SF2*) is positioned on chromosome 19 (19p12) and encodes a regulatory protein involved in very-low-density lipoprotein (VLDL) secretion, which is expressed in the intestine, renal system, and the liver [70]. According to some genetic studies, there is a correlation between the nonsynonymous variant in *TM6SF2* (E167K, rs58542926) with the content of triglycerides in the hepatic tissue, as well as its implications in cardiovascular diseases. The rs58542926 C > T polymorphism is associated with increased liver disease risk and decreased cardiovascular disease event risk [71]. The participation of *TM6SF2* in the development of NAFLD has been validated through clinical and epidemiological investigations. Recently, it has been shown that *Tm6sf2* plays a major role in stimulating hepatic fibrosis and HCC in mouse models [72]. According to a study conducted by Kozlitina et al., the frequency of the TM6SF2 variant encoding p.Glu167Lys (this mutation alters glutamate at residue 167 to lysine) was 7.2% in Caucasians, 4.7% in Hispanics, and 3.4% in African Americans [70].

The TM6SF2 protein has a critical function in the hepatic release of VLDL and the clearance of lipids in the intestines [73]. The *TM6SF2* gene E167K variant (rs58542926) involves a guanine-to-adenine substitution at nucleotide position 499, resulting in a glutamate-to-lysine change at amino acid position 167 (E167K) [74].

The hepatic synthesis of lipids containing PUFAs is impaired in *TM6SF2* E167K gene variant carriers, resulting in a shortage of polyunsaturated PCs and excess polyunsaturated FFA in hepatic tissue. The aforementioned alterations can explain the different phenotypic characteristics observed in *TM6SF2* E167K carriers, such as steatosis, absence of circulating triglyceride levels, and a heightened risk for steatohepatitis [57]. A strong correlation was found among the *TM6SF2* rs58542926 polymorphism and NAFLD presence in the overall population after a meta-analysis of 12 studies [75]. It is noteworthy that this polymorphism activates hepatic tissue damage and disrupts lipid metabolism modulation [76]. This association was significant in pre- and post-menopausal women rather than men [73]. EASL hepatology guidelines further validate that individuals who carry the aforementioned variant exhibit increased fat accumulation in the liver and are at heightened risk for developing NASH [77].

The *TM6SF2* variant p.E167K (rs58542926) alters the biosynthesis of PUFAs and leads to a reduction in PUFA levels in polyunsaturated PCs and TGs in the hepatic tissue. Additionally, it increases the polyunsaturated free fatty acids (FFAs). This alteration also reduces the overall concentration of FFAs, thereby embedding VLDL synthesis [78]. Rs58542926 exhibits proinflammatory properties, often associated with increased serum aminotransferase activity, while showing no correlation with GGT levels [79]. Histologically, two cohort studies involving 1074 patients linked the presence of rs58542926 with both hepatic steatosis and fibrosis (OR = 1.38, 95%CI = 1.019–1.865) [80]. Another examination of SNPs in 320 NAFLD patients revealed heightened risks associated with steatosis grade ≥ 2 (OR = 1.90) and fibrosis grade ≥ 3 (OR = 2.35) after adjustments for age, sex, body mass index, type 2 diabetes, and statin use [79]. Sookoian et al. have demonstrated significant associations with the risk of NAFLD, disease intensity, and degree of steatosis while noting weaker correlations with inflammation, NAFLD activity score, hepatocellular ballooning, and fibrosis [81]. The *TM6SF2* rs58542926 C > T minor allele is a potential marker for steatosis, MASH, and advanced fibrosis in MASLD patients. Additionally, homozygote carriers of this allele show increased HCC risk in MASLD patients [80]. The impact of this allele appears to extend to cirrhosis and predisposition to HCC, as evidenced in both unadjusted and adjusted models that consider factors such as age, sex, diabetes, obesity, and fibrosis [80,82].

### 2.3. GCKR

The glucokinase regulator (*GCKR*) gene is responsible for producing the glucokinase regulatory protein (GKRP), which serves as a specific inhibitor of the glucose-metabolizing enzyme known as glucokinase (GCK) in hepatocytes. Through GWAS, a prevalent coding variant within GCKR has been linked to various metabolic characteristics. In-depth functional assessments have revealed several molecular pathways associated with GKRP malfunction [83]. A meta-analysis by Zain et al. provided data of a robust correlation between the GCKR rs780094 and the risk of NAFLD [84].

The *GCKR* p.P446L (rs780094) variant diminishes the inhibitory impact when exposed to fructose-6-phosphate, leading to heightened glycolysis and glycogen synthesis, along with a simultaneous stimulation of de novo lipogenesis [85]. Additionally, based on a meta-analysis, rs780094 has been suggested as a potential index for NAFLD in homozygotes [84].

Regarding the *GCKR* gene variant rs1260326, it is linked to increased hepatic fat accumulation through glucokinase dysregulation, promoting glucose uptake and lipogenesis in the liver [86].

### 2.4. MBOAT7

The *MBOAT7* locus rs641738 C > T variant correlates with decreased phosphatidylinositol levels in hepatocytes and circulation [87]. This reduction is linked to an elevated risk of NAFLD, inflammation, fibrosis, and HCC [82,87]. This variant decreases *MBOAT7* gene expression in the liver, leading to reduced arachidonic acid binding to lyso-phosphatidylinositol [53].

Research by Donati et al. indicates that the MBOAT7 rs641738 T allele, linked to diminished MBOAT7 expression, may increase HCC susceptibility without cirrhosis. These findings underscore the crucial influence of genetic predisposition on HCC risk [82]. The rs641738 MBOAT7 variant is prevalent in European Americans (minor allele frequency [MAF] = 0.42, indicating that 42% of alleles in this population are the less common variant), followed by African Americans (MAF = 0.34) and Hispanics (MAF = 0.33). This variant decreases *MBOAT7* gene expression in the liver, leading to reduced arachidonic acid binding to lyso-phosphatidylinositol [53].

A study conducted by Thangapandi et al. indicated that *Mboat7* knockout mice on a specific diet exhibited elevated hepatic triglyceride levels compared to those on a standard diet [88]. Fibrotic changes were noted in the knockout mice, with negligible inflammation effects. These alterations were also seen in biopsy-confirmed NAFLD patients possessing the rs641738 *MBOAT7* variant, suggesting hepatic fibrosis development occurred independently of inflammation [88]. Additional results demonstrated parallels between *Mboat7* knockout mice and NAFLD patients with the homozygous *MBOAT7* variant, revealing analogous lipid composition changes in both models [88].

### 2.5. HSD17B13

Moreover, there are some genes that appear to exhibit hepatoprotective action. For instance, a comprehensive exome-wide study involving over 70,000 individuals showed that individuals homozygous for the minor variant rs72613567 T/A of *HSD17B13* (Hydroxysteroid 17-beta retinol dehydrogenase 13) experience a reduced risk of hepatic injury associated with *PNPLA3* and mitigate the risks of NAFLD, NASH, and cirrhosis by 30% and 49% accordingly [89]. More specifically, HSD17B13 expression, which is a protein associated with hepatocytes and lipid droplets, is significantly elevated in NASH patients compared to healthy individuals [90]. Members of the HSD17B family facilitate NAD(P)H/NAD(P)+-dependent oxidoreductase activity, influencing the balance of steroid forms [91]. The rs72613567 variant is prevalent in NAFLD patients and exhibits high linkage disequilibrium with another variant, which introduces a duplicate adenine nucleotide, leading to diminished enzymatic function [90]. Studies show that rs72613567 correlates with a reduced risk of ALD, NASH, and chronic liver disease, and this association is attributed to its role in mitigating liver damage, as reflected in lower serum ALT and AST levels [89,90]. The results highlight the protective role of rs72613567 in reducing disease advancement from steatosis [89,90]. In conjunction with *PNPLA3*, *TM6SF2*, or *MBOAT7* variants, rs72613567 improves predictive accuracy for NASH severity and advanced fibrosis [91].

### 2.6. Other Genes

Several other genes have also been found to be associated with NAFLD, such as *SERPINA1*, *APOE*, *APOB*, *IL28B*, *MERTK,* and *HFE*.

More specifically, the Serpin Family A Member 1 (S*ERPINA1* rs17580) gene correlates with liver cirrhosis, inflammation, and fibrosis [92]. The heterozygous presence of a1-antitrypsin mutations from the *SERPINA1* gene elevates the risk of chronic liver disease and cirrhosis, and a1-Antitrypsin deficiency is a prevalent genetic disorder impacting both lung and liver [93]. Furthermore, the *SERPINA1* Pi*Z allele is linked to higher liver-related mortality rates [94]. Recent research indicates a connection between *SERPINA*1 Pi*Z and increased disease severity and cirrhosis risk in NAFLD and ALD patients [95]. Notably, this correlation exists independently of *PNPLA3*, *TM6SF2*, and *MBOAT7*, with a stronger association for NAFLD/ALD cirrhosis than other risk factors [89,96].

Regarding *ApoE*, it plays a role in the synthesis of lipoproteins, encoding a key protein component [97]. NAFLD is marked by elevated plasma ApoE levels, irrespective of ApoE genotypes [98]. Elevated plasma ApoE may influence VLDL metabolism and enhance atherosclerosis risk in NAFLD [98]. Additionally, in a study conducted in mice by Lu et al. 2020, they found that *ApoE* deficiency disrupts the AMPK/mTOR pathway, reduces autophagy, and impairs hepatic mitochondrial function, resulting in NAFLD and suggesting that ApoE may regulate the AMPK/mTOR pathway through the modulation of hepatic mitochondrial function in NAFLD [97]. This investigation revealed that *ApoE*−/−-*HFD* mice demonstrated early stages of hepatic fibrosis, a predominant factor in the development of advanced HCC [71,97,99].

A multi-trait statistical genetics approach identified twelve DNA variants linked to cirrhosis risk, including seven novel variants [100]. These variants, when combined into a polygenic risk score, pinpointed a population subset at significantly elevated risk from obesity or excessive alcohol intake. Additional genetic loci implicated in cirrhosis progression risks include *HMBS*, *MAFB*, *CENPW*, *EFNA1,* and *SERPINA1*, among others [100].

Whole-exome sequencing has revealed rare variants potentially linked to NAFLD [101,102]. Similar to ApoE, ApoB is essential for cholesterol transport in lipoproteins [103]. The rare p.K2240X variant of APOB has been correlated with hepatic steatosis and may also relate to cirrhosis and liver cancer, based on extensive studies [103].

Furthermore, genetic and epigenetic biomarkers have gained significant interest recently [104]. Telomerase reverse transcriptase (TERT) promoter mutations are the most prevalent form of molecular alteration occurring in HCC and can be assessed via cfDNA [104]. Akuta et al. identified the TERT C228T mutation in 63.9% of NAFLD-HCC patients through cfDNA analysis, even with normal alpha-fetoprotein and des-carboxyprothrombin levels [105]. A 2021 study established the diagnostic significance of TERT mutations in the identification of HCC linked to NAFLD, enabling the early detection of HCC even in cases where alpha-fetoprotein levels are within normal range [105]. Thus, TERT mutations may facilitate the early detection of the disease and consequently improve the diagnosis.

**Figure 3:**
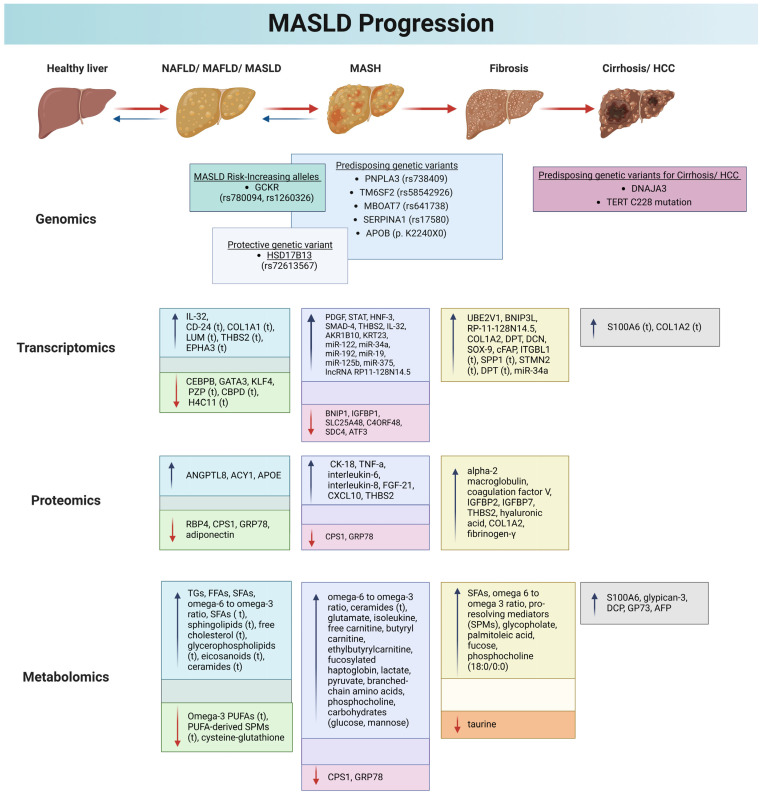
Key findings from the main omics technologies during MASLD progression. Blue arrows indicate upregulation, while red arrows indicate downregulation. (t) denotes hepatic tissue. Created in BioRender, https://BioRender.com/h50f514 (accessed on 11 February 2025).

Figure 3 summarizes the main gene mutations that have been related with the various stages of MASLD and its progression.

## 3. Transcriptomics and MASLD

Transcriptomics profiling provides comprehensive information about the transcription level of the human genome and gene structure and function while it clarifies the gene expression regulation and the genome plasticity [106]. Notably, transcriptomics has the potential to reveal significant alterations in biological processes that can initiate various human diseases, thus providing new avenues for the comprehensive analysis of the underlying mechanisms of diseases, as well as for their diagnosis and treatment [106].

Transcriptomics includes the quantitative evaluation of all the RNA molecules (called transcripts) expressed inside the cell, along with their corresponding transcriptional dynamics. Regarding the study of NAFLD, the NAFLD transcriptome constitutes the intermediate step while bridging the gap between the genome and the steatotic, inflammatory, and fibrogenic phenotypes [46].

Methods like RNA sequencing and microarrays elucidate the functions of transcriptional components [46]. RNA microarrays have revealed that there is an upregulation of genes related to lipid metabolism, acute phase regulators of insulin sensitivity, cellular division, DNA and tissue repair, extracellular matrix organization, immune function, cellular adhesion and migration, signal transduction, P53 signaling, and cancer progression in NAFLD and NASH [46,107,108,109,110,111,112]. On the other hand, genes related to mitochondrial function and glucose, oxidative, fatty acid, and amino acid or protein metabolism are downregulated [46,107,112,113].

Single-cell RNA sequencing (scRNA-seq) and spatial transcriptomics are applied to NAFLD and other liver diseases. Lin et al. highlighted how these methods provide insights into cellular heterogeneity and the spatial arrangement of cells, which are crucial for understanding disease mechanisms in NAFLD progression [114]. Single-cell techniques enable the identification of rare cell types and the tracking of cell state transitions, while spatial transcriptomics provides insight into the spatial organization of cells within tissues. These technologies have advanced the study of liver biology by revealing crucial information about liver homeostasis, development, regeneration, chronic liver disease, and cancer [114].

Govaere et al. used RNA sequencing in order to analyze gene transcription changes in hepatic tissue samples from 206 patients with histologically characterized NAFLD. Unsupervised clustering categorizes NAFLD by disease activity and fibrosis, considering age, AST levels, type 2 diabetes, and the *PNPLA3* rs738409 variant. The study used high-throughput whole-genome liver transcriptomics through RNA-seq to stratify NAFLD based on disease activity and fibrosis stage, and it identified a 25-gene liver transcriptome signature for advanced disease stages [115]. Additionally, this 25-gene signature was validated through logistic modeling in an independent cohort (*n* = 175), and the results were cross-referenced with publicly available single-cell RNA-seq data to understand the intrahepatic cell-type contributions during NAFLD progression [115]. Using high-throughput RNA-seq, that study stratified patients with NAFLD based on disease activity, fibrosis stage, and other clinical and genetic factors. This revealed dynamic changes in macrophage populations during the progression of NAFLD, highlighting the role of immune cells in disease progression. The researchers demonstrated that changes in RNA expression correlated with changes in hepatic protein levels, providing further validation of the identified biomarkers and their clinical relevance [115].

In another study, which included 306 patients with histologically characterized NAFLD, 4730 proteins were detected, and further transcriptomic analysis of paired hepatic tissue samples was carried out [116]. More specifically, this study developed a proteo-transcriptomic map of 31 markers after a correlation between the proteomic profiles for active steatohepatitis and advanced fibrosis with hepatic transcriptomics. scRNA-seq enables the identification of specific hepatic cell types likely contributing to these proteomic alterations during the disease progression. Logistic regression models established a composite framework integrating four proteins (ADAMTSL2, AKR1B10, CFHR4, and TREM2) alongside factors such as body mass index and the presence of type 2 diabetes to detect individuals at risk of developing steatohepatitis [116].

Transcriptomics has also been used in combination with spatial metabolomics for investigating the hepatoprotective effects of wedelolactone and demethylwedelolactone, which are the two major coumarin compounds derived from *Eclipta prostrata* L. [117]. This study revealed that steroid biosynthesis and fatty acid metabolism are primarily involved in the hepatoprotective effects of wedelolactone instead of demethylwedelolactone, thus unveiling a distinct mechanism in ameliorating NAFLD [117]. In a study conducted by Han et al., qRT-PCR was used to validate several key genes identified through machine learning algorithms as potential diagnostic biomarkers for NAFLD. RNA isolation from peripheral blood samples followed by qRT-PCR provided a means to assess gene expression related to immune cell infiltration, which is linked to NAFLD progression. In NAFLD patients, a downregulation of the expression of *CEBPD*, *H4C11*, *GATA3*, and *KLF4* was shown [118].

In NAFLD tissues, *CD24*, *COL1A1*, *LUM*, *THBS2*, and *EPHA3* genes are overexpressed, while PZP mRNAs are underexpressed according to large databases for differential gene expression [110].

Baselli et al. highlighted the significance and the overexpression of the interleukin-32 gene in NAFLD patients, as well as its potential usefulness as a candidate biomarker and an NAFLD-related cytokine. This study, which included 125 individuals with obesity, emphasized that the *PNLA3* I48M variant was a significant modifier of the liver transcriptome and that interleukin-32 was the most strongly upregulated transcript in severe NAFLD (defined as the presence of steatohepatitis, NAFLD activity score ≥ 4 or fibrosis stage ≥ 2). Interleukin-32 circulating levels are associated with hepatic expression and are upregulated in patients with NAFLD; thus, interleukin-32 is a candidate biomarker for the non-invasive evaluation of NAFLD and may be targeted for treatment [119].

The transcriptomic profile of NASH is characterized by the upregulation of genes associated with the *PDGF*, *SMAD-4*, *STAT,* and *HNF-3* pathways and the downregulation of *BNIP1* and *IGFBP1* [120]. Additionally, there was a downregulation in *SLC25A48* and *C4ORF48* [111], as well as in *SDC4*, *ATF3* [107] (various inflammatory inhibitors and genes involved in amino acid metabolism and the scavenging of ROS).

Starman et al. conducted a study in order to elucidate prospective molecular biomarkers that may facilitate the differentiation of steatohepatitis from steatosis [112]. The Gene Ontology (GO) analysis elucidated that the genes exhibiting downregulation in steatohepatitis predominantly participate in metabolic processes, while the genes that were upregulated in samples of steatohepatitis were linked to the progression and proliferation of cancer [112]. In samples from surgical liver resections, there were 39 genes, while in percutaneous liver biopsies, there were 30 genes, which exhibited significant upregulation in steatohepatitis. Furthermore, there was a notable elevation in AKR1B10 protein expression in steatohepatitis following the immunohistochemical examination of human hepatic tissue [112]. These demonstrated that KRT23 and AKR1B10 exhibited significant differential expression in steatohepatitis in comparison to steatotic and healthy liver, suggesting their potential utility as biomarkers for steatohepatitis and as indicators of progression to HCC [112].

Gerhard et al. conducted a sequencing-based analysis of mRNA profiles in hepatic tissue samples derived from individuals exhibiting normal liver histology (*n* = 24), lobular inflammation (*n* = 53), and advanced fibrosis (*n* = 65) [121]. A complementary approach was employed to assess a hepatic stellate cell line and examine the hepatic expression of specific mRNAs, along with identifying common transcriptional patterns in cirrhosis stages [121]. This analysis encompassed 3820 and 2980 transcripts in cases of lobular inflammation and advanced fibrosis, respectively, in contrast with normal histology, with 176 genes being uniquely associated with fibrosis. These identified genes were found to participate in pathways related to cytokine–cytokine receptor interaction, PI3K-Akt signaling, focal adhesion, and extracellular matrix–receptor interaction. The study pinpointed 34 differentially expressed transcripts between lobular inflammation and fibrosis, with some also elevated during hepatic stellate cell activation. A cohort of 16 genes from a prior NASH fibrosis/cirrhosis study was replicated, with several linked to advanced fibrosis/cirrhosis from hepatitis or alcohol in humans [121]. The dysregulation of mRNA expression was found to be linked to inflammation and fibrosis in NASH and with advanced fibrosis in NASH, exhibiting a distinct array of molecular alterations shared with other cirrhosis etiologies [121].

Moreover, Kozumi et al., after performing a transcriptomic analysis, illustrated that serum thrombospondin 2 expression (TSP-2) encoded by the *THBS2* gene was upregulated in NASH and that levels of TSP-2 were markedly linked to progressive fibrosis in individuals with NAFLD [122]. Furthermore, THBS2 exhibited positive correlations with inflammation, ballooning, serum aspartate aminotransferase, hyaluronic acid levels, and NAFLD Fibrosis Score (NFS). Its association with processes like extracellular matrix synthesis, platelet activation, caspase-mediated cleavage of cytoskeletal proteins, and immune cell infiltration was also observed. Subsequently, serum TSP-2 levels were assessed in 213 NAFLD patients, highlighting significantly elevated levels in NASH compared to NAFL, with a corresponding increase based on fibrosis stage [122]. Independent predictors of NASH and advanced fibrosis were identified as serum TSP-2 levels and platelet count. Notably, serum TSP-2 levels could aid in stratifying NAFLD patients based on the risk of hepatic complications, such as liver cancer and decompensated cirrhosis events [122]. Notably, HCC was exclusively detected in patients exhibiting elevated levels of TSP-2, indicating a probable utility of this marker for monitoring purposes [122].

The differential expression of microarray signals, such as UBE2V1, BNIP3L, and RP11-128N14.5, which are associated with oxidative stress, inflammation, apoptosis, and fibrogenesis, demonstrate upregulation in NAS ≥ 5 and fibrosis stages 3–4 [123]. Li et al. identified 45 differentially expressed genes (43 upregulated and 2 downregulated genes) and 10 hub genes, notably decorin (DCN), dermatopontin (DPT), and SRY-box transcription factor 9, from GEO databases in cirrhotic liver specimens. The upregulation of DCN, DPT, and SOX9 exhibited a positive correlation with fibrosis severity, though variations in their associations with the 5-year survival rate of HCC patients may exist [124]. The expression and levels of notable genes, such as *ITGBL1*, *cFAP*, *SPP1* (which encodes osteopontin), *STMN2* [which encodes SCG10 (Superior Cervical Ganglia-10 Protein)], and *DPT,* are elevated in accordance with the increase in liver fibrosis stages [125,126,127,128,129], while the expression levels of *S100A6* are heightened in primary human HCC and cholangiocarcinoma [121,130].

In a study conducted by Sun et al., bioinformatic techniques were employed to elucidate pathways and genes associated with NAFLD progressions, while three machine learning models were integrated to develop a gene signature for risk stratification [131]. Furthermore, bulk RNA sequencing, scRNA-seq, and whole-exome sequencing (WES) data were thoroughly analyzed to uncover genomic changes and altered pathways across different molecular subtypes of NAFLD. The results revealed two distinct subtypes of NAFL, one of which exhibits an inflammatory profile and fibrotic potential similar to NASH. The developed gene signature effectively distinguishes advanced NAFLD samples. *COL1A2*, a key gene associated with NAFLD progression, is predominantly expressed and upregulated in fibroblasts linked to HCC and is significantly correlated with the epithelial–mesenchymal transition (EMT) and angiogenesis in various cancers [131]. Furthermore, the β-catenin/COL1A2 axis may play a crucial role in fibrosis severity and inflammatory responses during NAFLD-HCC progression [131].

### 3.1. Micro-RNAs and Non-Coding RNAs

Micro-RNAs (miRNAs) and long non-coding RNAs (lncRNAs) play a crucial role in epigenetic and post-translational activities; thus, they influence transcriptional activity in various diseases, including NAFLD [132,133]. These epigenetic modifiers are extensively studied through differential miRNA expression panels [134,135], highlighting specific roles of miRNA subtypes in NAFLD [136,137]. Circulating miRNAs have the potential to function as non-invasive biomarkers for the evaluation of steatosis, liver stiffness, and hepatic fat content, which are critical for the diagnosis of MASLD [138]. Notably, miR-122 represents approximately 70% of hepatic microRNA, and human expression studies indicate that hepatic miR-122 levels rise in early NAFLD and then gradually decline with NASH progression and fibrosis advancement [139]. Elevated miR-122 levels are observed in simple steatosis compared to healthy livers and in severe steatosis versus mild steatosis [140,141]. Conversely, miR-122 levels are diminished in individuals with obesity and NAFLD relative to non-steatotic controls, in NASH relative to simple steatosis or healthy controls, and in severe fibrosis compared to mild fibrosis [140,141,142]. miR-122 is linked to glucose and lipid metabolism, and its inhibition lowers plasma cholesterol and enhances liver steatosis without adverse liver effects [143]. The upregulation of miR-122 inhibits cell growth and enhances cancer cell chemosensitivity to antitumor agents [144,145]. miR-122 may represent a potential therapeutic target for HCC through the modulation of its expression [146].

In steatohepatitis, circulating miRNAs have emerged as biomarkers that reflect the severity of the disease [147]. Cheung et al. observed a significant 63% decrease in the hepatic expression of miR-122 in patients afflicted with NASH relative to control subjects, thereby identifying 46 distinct miRNA species exhibiting differential expression, along with their respective targets [135]. Pirola et al. showed that among four circulating miRNAs analyzed, miR-122, miR-192, miR-19, miR-125b, and miR-375 were upregulated either in NAFLD or NASH, while miR-122 was downregulated in NASH compared to simple steatosis [140]. Additionally, miR-301a, miR-34a, and miR-375 appear to affect carbohydrate and lipid metabolism, showing associations with NAFLD severity and the development of HCC [134]. A meta-analysis conducted by Liu et al. on miR-34a reported an AUROC of 0.78 in differentiating simple steatosis from steatohepatitis [147].

Among these, predictive models utilizing miRNAs show promise for robustness and reproducibility since miRNAs can withstand multiple freeze–thaw cycles and long-term storage without degradation [148]. Multiple studies have identified miR-122 as a promising diagnostic biomarker, suggesting that miR-122—either alone or in combination with other miRNAs like miR-1290, miR-27, miR192, miR-34, and miR-99a—can reliably predict the presence of NAFLD [140,147,149].

In a study conducted by Tobaruela-Resola et al., data from 55 participants with steatosis (MASLD group) and 45 without steatosis (control group) were analyzed, while various health metrics and lifestyle factors were assessed. RT-PCR was employed to measure circulating miRNA levels. Elevated levels of miR-122-5p, miR-151a-3p, miR-126-5p, and miR-21-5p were significantly increased in the MASLD group. These miRNAs exhibited a significant correlation with steatosis, liver stiffness, and hepatic fat content [138].

Another model that integrates miR-122, miR-192, miR-21, and CK-18 fragments shows promise in differentiating NASH from NAFL [150].

Serum levels of miR-379 exacerbate cholesterol-induced lipotoxicity by disrupting insulin-like growth factor 1 signaling pathways [151]. The concentrations of circulating miR-21, along with their hepatic expression, may exhibit elevations in individuals with NAFLD as well as in murine models of the condition [152]. Consequently, miRNAs represent promising therapeutic targets that could yield innovative approaches for the clinical management of MASLD.

Various diagnostic assays/algorithms have been developed that incorporate miRNAs together with other proteins. Notably, NIS4™, which integrates miR-34a, alpha-2-macroglobulin, and chitinase-3-like protein 1, exhibited superior performance in diagnosing advanced NASH (NAS ≥ 4) and significant fibrosis (F ≥ 2) compared to other tests like FIB4, ELF, or Fibrometer, although its efficacy may still be suboptimal [153].

### 3.2. Long Non-Coding RNAs (lncRNAs)

lncRNAs, defined as non-coding RNAs exceeding 200 nucleotides in length, play a critical role in the regulation of transcriptional processes pertaining to protein-coding genes [154]. Furthermore, lncRNAs hold potential as both diagnostic and therapeutic targets in the context of NAFLD.

An examination of comprehensive hepatic RNA profiles has indicated the overexpression of 535 lncRNAs and 760 messenger RNA (mRNA) species in NAFLD, with 1200 lncRNAs and 725 mRNAs being underexpressed, primarily participating in small molecule and organic acid metabolic pathways [155]. Functional transcriptome analyses focusing on 4383 lncRNA species by Atanasovka et al. have identified heightened levels of hepatic lncRNA RP11-484N16.1 in NASH, which shows associations with NASH grade, lobular inflammation, and NAFLD activity score (NAS) and affects hepatic growth and viability upon knockdown in vitro [156]. The upregulation of lncRNA RP11-128N14.5, which is associated with the diagnosis of NASH, has been validated through whole-serum transcriptome analysis and is elevated in NAS ≥ 5 [123]. Additionally, the profiling of transfer RNAs (tRNAs) demonstrates varying expression levels of different anticodons for lysine, glutamate, and aspartate in cirrhosis, as well as distinctions in mitochondrial and amino acid tRNAs across cirrhosis, NAS, NASH cirrhosis, and normal samples [134]. Finally, this study examined the prevalence of 392 small RNA molecules, including ribosomal, small nuclear, and nucleolar, among healthy individuals, NASH patients, and individuals with cirrhosis.

Additionally, comprehensive genetic investigations conducted in murine models and primary hepatocyte cultures indicate that the lncRNA known as the regulator of hyperlipidemia (lncRHL) serves to activate the lncRHL/heterogeneous nuclear ribonucleoprotein U (hnRNPU)/brain and muscle aryl hydrocarbon receptor nuclear translocator (ARNT)-like protein 1 (BMAL1)/microsomal triglyceride transfer protein (MTTP) signaling pathway, thereby unveiling novel molecular mechanisms that regulate lipid homeostasis within hepatic and circulatory systems [157]. The interaction of lncRNA with hnRNPU results in the transcriptional activation of BMAL1, which consequently leads to the inhibition of VLDL secretion from hepatocytes [157]. Additionally, the lncRNA nuclear-enriched abundant transcript 1 (NEAT1) intensifies FFA-induced lipid accumulation in the liver through modulation of the c-Jun N-terminal kinase (JNK)/sterol regulatory element-binding protein 1c (SREBP-1c) axis [158]. Notably, the levels of sorafenib resistance-associated lncRNA (lncARSR) are found to be elevated in both the serum and hepatic tissues of patients diagnosed with NAFLD [158]. Moreover, lncRNA (MRAK052686) exhibits a correlation with the antioxidant factor Nrf2, and its downregulation appears to facilitate the progression of steatosis [159]. Collectively, this evidence indicates that the pathogenesis of MASLD is intricately linked to the dysregulated expression of lncRNAs.

### 3.3. Circular RNAs (circRNAs)

Circular RNAs (circRNAs) are unique RNA molecules with covalently closed structures, known for their stability, diversity, and conservation [160]. A review by Zeng et al. highlights the biogenesis, properties, and functions of circRNAs and examines circRNA expression in NAFLD by analyzing seven sequencing datasets [161]. Notably, there is a potential for targeting circRNAs and competing endogenous RNA networks for NAFLD therapy using gain-of-function and loss-of-function strategies [161].

circRNAs, which are non-coding RNAs associated with lipid metabolic processes, represent promising therapeutic targets for hepatic disorders [161]. Mice subjected to a high-fat diet exhibit dysregulated expression of CircStearoyl CoA desaturase 1 (circSCD1), which influences the extent of steatosis and exacerbates MASLD through the JAK2/STAT5 signaling pathway [162]. Bioinformatic analyses revealed a genome-wide dysregulation of circRNAs correlated with hepatic steatosis [163]. In both MASLD-afflicted murine models and in vitro cellular systems, circ_0057558 modulates Rho-associated protein kinase 1/AMPK signaling by interacting with miR-206, thereby facilitating the progression of MASLD [164]. Thus, the dysregulation of circRNAs is potentially associated with the pathophysiology of MASLD.

Figure 3 summarizes the main RNA molecules and their regulation during MASLD progression.

## 4. Proteomics and MASLD

The term proteomics refers to the investigation of the entirety of proteins within an organelle, cell, tissue, or organism [165]. The Human Liver Proteome Project, initiated in China in 2003, served as a precursor to the HPP and facilitated the elucidation of liver protein expression profiles and protein–protein interactions (PPIs) [166,167,168].

Proteomics is crucial for understanding the dynamic complexities of human biology, which cannot be fully predicted from the genome alone [168]. Recent advances in proteomic technologies and computational sciences now allow for the unprecedented exploration of protein function and interactions within the human body [168].

Initiatives like the π-HuB project aim to harness these advancements to improve disease diagnosis, uncover therapeutic targets, and enable precision medicine through proteomics-driven healthcare innovation [168].

Younossi et al. studied serum protein profiles in NAFLD in a cohort of 98 individuals with obesity [107]. In that study, 91 individuals were diagnosed with NAFLD (12 presenting with steatosis alone, 52 displaying steatosis along with nonspecific inflammation, and 27 identified with NASH), whilst 7 participants without NAFLD comprised the study control group. Among the 300 detected protein peaks, 16 showed significant differences across groups. While specific proteins were not identified, their masses matched 1440 serum proteins, highlighting fibrinogen γ as a potential fibrosis [107].

A study by Bell et al. employed an ion-intensity-dependent, label-free, quantitative proteomics technique to identify proteins showing significant changes between NAFLD and NASH characterized by advanced fibrosis [169]. That study used liquid chromatography–tandem mass spectrometry (LC-MS/MS) to investigate the serum protein profiles of 69 patients categorized into those with simple steatosis, NASH, or NASH with advanced fibrosis, as well as 16 control individuals with obesity. A panel of six proteins (including fibrinogen β chain, retinol binding protein 4, serum amyloid P component, lumican, transgelin 2, and CD5 antigen-like) successfully differentiates patient groups with a 76% overall success rate (AUROC: control 1.0, simple steatosis 0.83, NASH 0.86, NASH F3/F4 0.91. A panel of three proteins (component C7, insulin-like growth factor acid labile subunit, and transgelin 2) accurately categorizes 90% of patients with NAFLD or NASH F3/F4, achieving an AUROC of 0.91. Lastly, two proteins (prothrombin fragment and paraoxonase 1) distinguish control subjects from all NAFLD forms with 100% accuracy and an AUROC of 1 [169]. Among the above-referenced proteins, RBP4 protein was recognized for its markedly reduced expression associated with increased severity of NAFLD [169].

Another study investigated and compared the proteomic profiles of four distinct groups based on body mass index, namely (1) overweight NAFLD; (2) overweight control; (3) lean NAFLD; and (4) lean control group [170]. Lean patients with NAFLD present with intermediate metabolic disturbances compared to healthy and overweight NAFLD individuals, with altered plasma proteomic profiles suggesting potential biomarkers for the diagnosis and treatment of NAFLD in this population. Furthermore, the proteome of the plasma of the lean NAFLD patients, in contrast to the healthy group, showed that 62 proteins exhibited significant differences among the two groups (34 proteins were upregulated, while 28 were downregulated) [170].

Sanyal et al. developed serum proteomic models to diagnose NAFLD components and monitor disease activity and fibrosis progression. The models accurately reflected liver biopsy findings and identified key histological features, including at-risk NASH. Their responsiveness to changes highlights their potential for patient selection and monitoring in clinical trials, offering a promising non-invasive tool for NAFLD management. These findings represent an important step toward validating proteomic biomarkers for NAFLD [171].

Altomare et al. employed quantitative proteomic approaches to study protein changes in an in vitro NAFLD model (Hepatic G2 cells-hepatic hepatocellular carcinoma cells -HepG2 cells) and identify key affected pathways. The study identified 2482 proteins, with 17 downregulated and 36 upregulated proteins. Network analysis linked differential protein expression to processes like metabolic regulation, cell–cell adhesion, cytoplasmic organization, and cell invasion, suggesting increased proliferation driven by inflammation or oxidative stress. These findings highlight the potential of advanced bioanalytical methods for disease modeling and drug assessment in NAFLD [172].

Rodriguez-Suarez et al. investigated the changes in the proteome profile of patients with steatosis or steatohepatitis compared to controls. Their findings showed that 43 proteins exhibited significant changes (22 proteins were implicated in steatosis, while 21 were implicated in steatohepatitis) compared to controls. Furthermore, Western blot analysis was used to validate these changes in protein expression profiles in the three study groups. Carbamoyl phosphate synthase 1 (CPS1) and 78 kDa glucose-regulated protein (GRP78) were the two differentially expressed proteins, and they decreased from control to steatosis and NASH patients, representing potential candidate biomarkers [173].

Sveinbjornsson et al. analyzed circulating protein levels in NAFLD and cirrhosis patients using Icelandic and UK Biobank datasets. Key findings included increased IGFBP2 and THBS2 levels in cirrhosis compared to NAFLD and the general population, while ACY1 levels were higher in NAFLD in contrast to the general population. IGFBP2 was elevated in cirrhosis but reduced in NAFLD compared to the general population. The study confirmed the association of IGFBP2, IGFBP7, and other IGF-binding proteins with advanced fibrosis, consistent with prior research on NASH progression [174,175].

Additionally, contradictory epidemiological evidence exists regarding the relationship between NAFLD and SHBG levels [176]. Various NAFLD variants are connected with SHBG plasma levels, which have variable effects in relation to their impact on fat in the liver [175,176]. A recent study by Dong et al. indicated that the genetic predisposition for elevated SHBG levels is causally linked to a diminished risk of NAFLD, suggesting that high circulating SHBG serves as a protective element against NAFLD [177,178]. Furthermore, SHBG levels correlate positively with cirrhosis and advanced fibrosis in NASH [174,175]. In patients with advanced fibrosis, SHBG was elevated compared to those at fibrosis stages 0–2, with peak values in stage 4 [174].

Cytokeratin-18 is the most extensively investigated biomarker for the assessment of NASH [10]. CK-18 represents an intermediate filament protein fragment that is generated from the apoptosis of hepatocytes, thereby facilitating the association of its serum levels with the extent of hepatocyte injury for the purpose of appraising disease severity in connection to the histological alterations observed in steatohepatitis [41]. However, its clinical use is limited due to difficulties in measuring CK-18 and inconsistencies in cut-off values across studies [179]. The limited sensitivity of CK-18 as a standalone marker has prompted its use in combination with other biomarkers for improved accuracy. For example, combining CK-18 with interleukin-6 and adiponectin resulted in an AUROC of 0.90, with 85% sensitivity and 86% specificity to predict NASH [180]. These combinations are based on the inflammatory processes seen in patients with steatohepatitis, who often have obesity-related inflammation and altered adipokine profiles [181].

Regarding adiponectin, recent studies and meta-analyses have shown that lower plasma adiponectin levels are significantly associated with the presence and severity of NAFLD, suggesting hypoadiponectinemia as a potential risk factor for NAFLD [182,183,184].

Other potential biomarkers, like leptin, require further validation. Pro-inflammatory cytokines, such as chemokine (C-X-C motif) ligand 10 (CXCL10), TNF-α, and interleukin-8, show moderate accuracy in differentiating steatohepatitis from simple steatosis, with sensitivity and specificity ranging from 65% to 76% [185,186]. Combining TNF-α and interleukin-8 with pyroglutamate can boost sensitivity and specificity to 91% and 87%, respectively [186]. Fibroblast growth factor 21 (FGF-21), a liver-derived protein, has also been investigated, with an AUROC of 0.62 for diagnosing NASH [187]. When FGF-21 is combined with CK-18, the predictive values increase to 82% for positive prediction and 74% for negative prediction [187].

Younossi et al. investigated the use of phosphoproteomic biomarkers to predict histologic NASH and fibrosis [188]. Using reverse-phase protein microarrays, they analyzed visceral adipose tissue from NAFLD patients and developed clinical, proteomic, and combined models to evaluate kinase-driven signaling activity. The onset of NASH was associated with changes in insulin signaling, specifically the phosphorylation of GSK-3 and PKA subunits. However, phosphoproteomic markers were less effective in predicting fibrosis. This study highlights the involvement of the AKT kinase and insulin signaling pathways in NASH progression and identifies potential biomarkers for its prediction [188].

Another comprehensive analysis of the phosphoproteome of the hepatic tissue and the proteome of serum involving 67 individuals diagnosed with NAFLD through biopsy showed that the ASK1-MAPK pathway, triggered by IL-10, is relevant to hepatic fibrosis, as presented by its robust correlation with upregulated hepatic collagen levels. Moreover, alpha-2 macroglobulin and coagulation factor V in the serum displayed notable associations with hepatic collagen [5]. Thus, these pathways constitute potential therapeutic targets.

Trak-Smayra et al. investigated the serum protein profiles in individuals with obesity who were candidates for bariatric surgery, with the goal of identifying serum biomarkers associated with steatosis and NASH [189]. Their findings reported an association among the presence of liver lesions and increased levels of double-charged ions from alpha- and beta-hemoglobin subunits, indicating a significant rise in intensity corresponding to the severity of the hepatic lesions. Notably, these protein levels returned to normal following bariatric surgery [189].

Yuan et al. conducted a proteomic analysis on liver biopsies, revealing the presence of approximately 220 proteins exhibiting substantial variations in abundance between patients with NAFLD and those who were metabolically healthy despite obesity [190]. Proteins exhibiting elevated levels in NAFLD were found to be associated with the PPAR-signaling pathway and interactions with extracellular matrix receptors, while those with decreased levels were predominantly localized in mitochondria and involved in oxidative phosphorylation [190]. Concerning the complications of the disease, a study was conducted on the proteomic profile of hepatic tissues affected with NAFLD in mice that eventually developed HCC [190]. Protein S100A, which is known for its secretion by malignant cells and its function in enhancing cell proliferation and motility, was linked with advanced stages of HCC and poor outcomes [190].

Another study presented a multi-component classifier for hepatic steatosis using genomic, proteomic, and phenomic data based on data from 576 individuals with extreme obesity who underwent bariatric surgery and liver biopsy during surgery and showed superior predictive power in differentiating between NAFLD patients and healthy controls [191]. Protein biomarker discovery was conducted using the highly multiplexed SOMAscan proteomic assay, along with 19 clinical variables and the PNPLA3 rs738409 SNP genotype status, on a training set of 443 patients. These models integrated genomic data, focusing on the PNPLA3 rs738409 SNP genotype, along with biochemical measurements, including insulin, glucose, alanine aminotransferase, lipid profile, and proteomic info [191]. The extensive examination of the proteome revealed 1129 proteins, with 30 showing significant distinctions between the groups under comparison. Of note, proteins encompassed ami-noacylase-1, antithrombin III, SHBG, Galectin-3, and hepatocyte growth factor [191]. However, the effectiveness of such algorithms remains open to debate, given that simpler approaches, such as liver ultrasound or the fatty liver index (FLI), exhibit comparable or superior sensitivity and specificity [191]. Additionally, relying solely on the diagnosis of NAFLD may not offer adequate guidance for treatment decisions [191].

Another in vitro proteomics investigation by Lockman et al. investigated the distinct patterns of protein expression in human hepatoblastoma C3 cells, which were treated with a combination of energy substrates, such as lactate, pyruvate, octanoate, and ammonia, that lead to steatohepatitis with various characteristics like oxidative stress, impaired mitochondrial function, and altered glucose metabolism [192]. In that study, the protein extracts were trypsinized and analyzed on a capillary HPLC OrbitrapXL mass spectrometer. Among the 1327 proteins identified, 104 showed differential expression between the lactate (L), pyruvate (P), octanoate (O), and ammonia (N) (LPON-treated) cells and untreated cells, with 70 proteins of these being upregulated in LPON-treated cells. An analysis of functional enrichment indicated an increase in protein biosynthesis, coupled with the downregulation of histones H2A type 1-A, H1.2, H1.5, and H1.0I in LPON-treated cells. Additionally, there was enrichment observed in annotations related to lipid binding and proteins associated with cholesterol synthesis, uptake, and efflux. The heightened expression of aldo-keto reductase family 1, members C1 and C3 suggests an enhanced sterol metabolism and increased lipid peroxidation mediated by ROS. Based on the results, that study revealed that proteins associated with lipid metabolism (e.g., serum albumin, perilipin-2, APOAI, AKR1C1, and FABP1) are among the most significantly affected proteins [192].

Additionally, promising proteomic biomarkers for MASLD include angiopoietin-like proteins (ANGPTLs), which are part of a glycoprotein family with distinct tissue expression and regulation features and play crucial roles in insulin resistance, lipid metabolism, and hormonal regulation [193]. Several studies have revealed links between circulating ANGPTLs and NAFLD; although the findings are inconsistent [194,195,196]. A recent meta-analysis comprising 13 studies indicated a potential strong correlation between certain ANGPTLs and NAFLD, particularly showing significantly elevated levels of ANGPTL8 in NAFLD patients compared to controls [197]. The link between ANGPTL8 and the development of NAFLD has also been shown concerning the spectrum of disease progression, as individuals with mild to severe NAFLD tend to exhibit higher ANGPTL8 levels than those with moderate-severity NAFLD, underscoring the potential of this ANGPTL as an indicator for monitoring the disease across various stages [197].

Regarding HCC, notable biomarkers comprise alpha-fetoprotein (AFP), isoform of alpha-fetoprotein (AFP-L3), Des-γ-carboxyprothrombin (DCP), Glypican-3, and GP73, among others [198,199]. The GALAD score, incorporating age, sex, AFP, lectin-bound AFP, and DCP, emerges as the most dependable scoring system for HCC [200]. Research by Best et al. indicated that the GALAD score achieved a notable diagnostic performance, surpassing individual biomarkers like AFP and DCP [201]. Although the GALAD score holds promise, its utility is confined to advanced HCC, with limited applicability in early-stage monitoring. Ongoing investigations are exploring novel biomarkers, including genetic and epigenetic factors [200].

Additionally, Zhang et al. developed a reliable blood-based panel for the non-invasive diagnosis of MASH, termed N3-MASH, which includes a triad of parameters—C-X-C motif chemokine ligand 10 (CXCL10), cytokeratin 18 fragments M30 (CK-18), and adjusted body mass index (BMI). N3-MASH demonstrated high efficacy in distinguishing MASLD patients from healthy controls, achieving an AUROC of 0.954. Furthermore, N3-MASH identified MASH among MASLD patients with an AUROC of 0.823 in the discovery cohort [202].

Figure 3 summarizes the protein biomarkers and their distinct expression/regulation across MASLD progression.

## 5. Metabolomics and MASLD

Metabolomics can be defined as the large-scale study of small biological molecules present in cells, tissues, or organisms [203,204]. The entirety of these small molecules and their interplay within a biological system are recognized as the metabolome. The analysis of metabolites can offer an immediate overview of the physiological status of the cell, thus enabling metabolomics to serve as a direct indicator of an organism’s physiological condition. Notably, there exist measurable associations between the metabolome and other cellular components, such as the genome, transcriptome, proteome, and epigenome, enabling the estimation of metabolite levels in biological specimens [204]. A significant challenge in systems biology is the integration of metabolomics with other -omics data to enhance the comprehension of cellular processes.

Metabolomics in NAFLD focuses on analyzing the metabolic profile of individuals with NAFLD in order to understand the underlying molecular pathways associated with the disease. Metabolomics has provided valuable insights into modified metabolic mechanisms in NAFLD and NASH, offering a dynamic perspective on disease progression by studying the metabolic pathways and changes in the metabolite levels over time [205]. Several studies have indicated significant changes in amino acid metabolism and various facets of lipid metabolism, such as levels of circulating fatty acids, phospholipids, bile acids, and triglycerides [205]. Additionally, metabolomic studies have highlighted the associations between the circulating amino acids and steatohepatitis [205], emphasizing the role of impaired amino acid metabolism in insulin resistance, particularly in muscle cells.

The identification of metabolomic biomarkers in NAFLD is crucial for developing non-invasive diagnostic tests and monitoring treatment responses [205]. An imbalance in dietary lipid absorption, lipogenesis, and lipolysis may result in hepatic fat accumulation (NAFLD), subsequently leading to chronic inflammation, fibrosis, and liver cancer [203]. Studies on lipid composition have outlined specific transformations in the hepatic lipidome among individuals with NAFLD [206,207,208,209]. The hepatic levels of saturated fatty acids (SFAs), free cholesterol, sphingolipids and glycerophospholipids, glycerophospholipids, and eicosanoids rise, while omega-3 PUFAs and specialized pro-resolving mediators of PUFAs are decreased [210].

The accumulation of SFAs is positively linked to the severity of liver disease [210]. Within hepatocytes, SFAs prompt the release of pro-inflammatory cytokines by activating the pathway of toll-like receptor-4 and increasing ER stress and ROS while diminishing mitochondrial and peroxisome beta-oxidation by activating JNK and activating apoptosis via the pathway of TRAIL-2 signaling [210]. Concerning the non-parenchymal hepatic cells, SFAs promote the production and release of pro-inflammatory and pro-fibrotic cytokines from Kuppfer cells and prompt the pro-inflammatory M1 polarization of macrophages [210].

MUFAs and PUFAs also play a crucial role in NAFLD [210]. Palmitoleic acid (C16:1) and oleic acid (C18:1) are among the most extensively studied MUFAs, which exhibit lipotoxic characteristics, although at a reduced level in comparison to SFAs [210]. Hence, a greater MUFA/SFA ratio could potentially offer benefits attributed to the reduced ability of MUFAs to trigger ER stress and programmed cell death. Moreover, PUFAs include two categories, namely the omega-3s, e.g., eicosapentaenoic acid (EPA) and docosahexaenoic acid (DHA), and omega-6 fatty acids, e.g., arachidonic acid (AA) and dihomo-γ-linolenic acid [210]. The majority of omega-3 and omega-6 fatty acids are acquired through dietary intake. Conversely, the biosynthesis of highly unsaturated fatty acids like EPA, DHA, and AA involves distinct enzymes like elongase and desaturase enzymes [211]. In NAFLD, a significant disruption in the hepatic elongation of long-chain fatty acids is evident, resulting in an elevated omega-6 to omega-3 ratio and heightened flow in the omega-6 pathway [206,207,212].

The production of eicosanoids is prompted by the increased omega-6 levels and through the oxidation of enzymes (EPA, AA, and dihomo-γ-linolenic acid), alongside pro-inflammatory attributes like prostaglandins, thromboxanes, and leukotrienes, thereby inducing hepatic inflammation [213,214]. More specifically, the shift takes place at the expense of specialized pro-resolving mediators (SPMs), which principally serve to restore cellular functionality, thus mitigating chronic inflammation and fibrosis [213,214]. Moreover, the increased omega-6 to omega-3 ratio correlates with impaired FADS1 function, potentially impacting cell membrane phospholipid composition and leading to membrane insufficiency, cellular necrosis, and the extracellular accumulation of lipotoxic lipids, which may exacerbate liver tissue damage [209].

Several studies have proposed different diagnostic frameworks utilizing metabolomics and lipidomics alongside additional biochemical and clinical factors to establish non-invasive algorithms for diagnosing and staging NAFLD [215]. More specifically, the aim of these models is to diagnose advanced fibrosis, distinguish NAFLD from a state of health, discern between steatosis and NASH or NASH versus non-NASH, or recognize fibrosis irrespective of the severity. In another investigation, metabolomics and lipidomics data were integrated with genotype and biochemical data in order to develop an algorithm for distinguishing NASH from healthy individuals and NAFLD patients [216]. A total of 223 subjects were utilized for training this algorithm, with an additional 95 subjects for validation. Finally, the NASH ClinLipMet Score is a metabolic-based combination score that integrates the following five metabolites, namely glycine, isoleucine, glutamate, lyso-phosphatidylcholine 16:0, and phosphoethanolamine 40:6, as well as the PNPLA3 genotype and clinical variables, showing promise in distinguishing steatohepatitis from steatosis with an AUROC of 0.87 [216].

Another objective of the current research is to create non-invasive algorithms for distinguishing advanced (stage 3–4) from non-advanced (stage 0–2) hepatic fibrosis. In a notable study, a group of 156 individuals was first employed to formulate an algorithm following a metabolomic and lipidomic examination, incorporating 10 metabolites and lipids [217]. Within these components, six were precursors of steroid hormones based on cholesterol, showing significant reduction in advanced fibrosis, whereas one was the main conjugated bile acid, glycopholate, which was elevated in cases of advanced fibrosis. Furthermore, an additional component, the amino acid taurine, which is associated with bile acid conjugation, exhibited diminished concentrations in the context of fibrosis. Additionally, palmitoleic acid and fucose were recognized as elevated in advanced fibrosis. The algorithm resulting from these findings was authenticated in a twin and family group, as well as in a biopsy-confirmed NAFLD group, showing enhanced sensitivity and specificity when compared to other established indicators [217].

### 5.1. Applied Metabolomics in MASLD

Mass spectrometry has been widely used to measure metabolite markers of NAFLD, while the application of Nuclear Magnetic Resonance (NMR) Spectroscopy, though more limited, is steadily increasing. Both techniques are complementary, offering quantitative, targeted analysis of specific metabolites [218]. Robinson et al. utilized a curated, non-high-throughput NMR approach, aiming to improve the precision and detection limits of NMR quantitation to better identify metabolite trends in biopsy-confirmed NAFLD groups [218]. Given the growing potential to detect small molecule biomarkers for NAFLD, the study also assessed the feasibility of identifying biomarkers in retrospective cohorts using biobanked standard-of-care samples, evaluating their suitability for metabolomic analysis [218].

Li et al. have performed a study with the aim of identifying novel biomarkers for the non-invasive diagnosis of NAFLD [219]. That study investigated the time-related biochemical changes in mice sera induced by a methionine- and choline-deficient diet (MCD) and showed that many serum metabolite concentrations change between control and MCD-fed mice through NMR spectroscopy and principal component analysis (PCA). There were four potential biomarkers that could be used for the non-invasive diagnosis of various stages of NAFLD, namely serum glucose, lactate, glutamate/glutamine, and taurine [219]. These four metabolites were selected by hierarchical cluster analysis and artificial neural networks. In addition, the diagnostic accuracy of these four selected metabolites was verified by measuring their serum concentrations in healthy controls, NAFLD patients with steatosis, steatosis patients with necro-inflammatory disease, and NASH patients. Based on the results of this study, Li et al. proposed using the levels of serum glucose, lactate, glutamate/glutamine, and taurine for diagnosing NAFLD at various stages [219].

Kahlan et al. conducted a study in which they compared the metabolomic alterations through untargeted global metabolomic analysis in plasma specimens obtained from non-diabetic individuals with histologically confirmed hepatic steatosis in comparison with samples from healthy subjects matched for age and sex [220]. The researchers observed significant variations in bile acid levels, such as glycocholate, taurocholate, and glycochenodeoxycholate, as well as changes in glutathione-related biochemical parameters. In terms of long-chain fatty acid concentrations in plasma, they noted a decrease, while levels of free carnitine, butyryl carnitine, and ethylbutyrylcarnitine were elevated in individuals with NASH. Moreover, elevated levels of glutamyl dipeptides were observed alongside lower levels of cysteine-glutathione in NASH and steatosis. Additionally, there were elevated levels of branched-chain amino acids, phosphocholine, carbohydrates (glucose, mannose), lactate, pyruvate, and various unidentified metabolites. Through statistical analysis, a set of biomarkers was identified that effectively distinguished healthy controls from patients with NAFLD and healthy controls from those with NASH. However, the metabolomic profile did not allow for differentiation between hepatic steatosis and steatohepatitis [220].

Kozyra et al. applied untargeted metabolomics in order to analyze metabolites in a hepatic 3D spheroid system after inducing hepatic steatosis [221]. This 3D spheroid system successfully induces steatosis, demonstrates reversibility, and responds to antisteatotic compounds, showcasing its potential for drug discovery and the comprehension of the molecular mechanisms of fatty liver disease. Primary human hepatocyte spheroids sustain their viability and functionality for a duration of up to 21 days and have been used to generate an NAFLD model after exposure to elevated concentrations of insulin, monosaccharides, and non-esterified fatty acids (NEFAs) [221]. Subsequent to a seven-day period characterized by the accumulation of intracellular lipids, the manifestation of insulin resistance was observed within the spheroids by the 14th day, as indicated by the increased expression levels of phosphoenolpyruvate carboxykinase 1 (PCK1) and pyruvate dehydrogenase lipoamide kinase isozyme 4 (PDK4), alongside reduced phosphorylation of GSK3β. This study highlighted the importance of lipid droplets as potential therapeutic targets, emphasizing the need for interventions targeting key disease mechanisms like steatosis resolution for effective NAFLD treatment. Despite the lack of approved therapies, understanding the molecular events underlying NAFLD progression, particularly the role of lipid droplets, is crucial for developing effective pharmaceutical interventions [221].

Metabolomics and lipidomics may provide useful insights into distinguishing between steatosis and steatohepatitis and the prediction of hepatic fibrosis due to the strong link between MASLD and metabolic syndrome. Additionally, Caussy et al. showcased the efficacy of a predictive score, achieving an AUROC of 0.94, by incorporating a panel of serum metabolites in accurately predicting advanced fibrosis in NAFLD patients, surpassing FIB-4 and NFS [217].

### 5.2. Applied Lipidomics in NAFLD

Recent advances in mass spectrometry techniques have brought lipidomics to the forefront of translational research [222]. Lipidomics examines the lipid composition in cells, biological fluids, and tissues [223]. Lipids, among other metabolites, not only constitute the most prevalent components in the body’s circulation but also serve various crucial biological roles, including energy storage, cell signaling, and structural support for cell membranes [222]. The pertinence of lipid metabolism imbalance is of key importance for the development and progression of NAFLD.

Notably, there is evidence indicating that changes in the lipidome of liver tissue mirror lipid profile alterations in plasma, paving the way for utilizing a lipid profile as a biomarker for the histological characteristics of NASH [224]. Combining different circulating lipids may enhance the accuracy of diagnosis and risk assessment in NAFLD patients, allowing for the precise identification of those with NASH [225]. In a study involving NAFLD patients, a composite score derived from serum lipids, evaluated using nanoparticle-tracking techniques, and genetic variations successfully predicted fat fraction measured by MRI-PDFF [226].

Furthermore, in a cohort of individuals with biopsy-confirmed NASH, phosphatidylcholine levels, analyzed via LC-MS, were significantly linked to the severity of hepatocyte ballooning (Ogawa et al. 2020) [227]. Another study revealed positive correlations between phosphocholine (14:0/18:2) and phosphatidic acid (18:2/24:4) with the NAS score, while phosphocholine (18:0/0:0) exhibited a positive correlation with the stage of fibrosis [228].

Employing the same lipidomic method, Jambulingam et al. showed that a score integrating metabolic profile and lipoproteins effectively identified rapid fibrosis progressors, outperforming non-invasive markers [229].

Interestingly, ectopic fat depositions (e.g., myocardial and epicardial fat) exhibit a distinct lipid composition distinct from hepatic fat [230]. Notably, a higher presence of diacylglycerol and ceramide in ectopic fat deposits, measured using LC-MS, appears to be associated with a more pronounced lipotoxic impact overall [231]. Lipidomic strategies may offer deeper insight into metabolic alterations resulting from various treatments in NAFLD patients. A six-month regimen of PUFAs successfully altered the lipid profile of NASH patients, leading to reduced lipogenesis, ER stress, and mitochondrial dysfunction [232]. Similarly, weight loss in patients was accompanied by a notable decrease in circulating lysophospholipids [233]. Further investigations are warranted to assess how modifications in the lipidomic profile can translate into clinical outcomes.

### 5.3. Applied Glycomics in NAFLD

Glycomics falls within the realm of glycobiology, which focuses on the identification of glycan functions and their structural elucidation in specific biological contexts encompassing various cellular interactions (e.g., differentiation, development, morphogenesis, embryogenesis, immunity, infection, tumorigenesis, and metastasis) [234]. More specifically, glycomics refers to the thorough exploration of the glycome and the definition of the complete repertoire of glycans and glycoconjugates. The glycome displays a high degree of dynamism, influenced by factors like transcriptome, proteome, and environmental conditions, as well as cellular secretory mechanisms [235].

Glycans have the capacity to impact protein structure, protein–protein interactions, nutrient storage, and sequestration regulation [236]. Moreover, they play a role in maintaining cell stability and facilitating intercellular communications [236]. Glycosylation involves the synthesis of glycoconjugates and can occur intracellularly or extracellularly [235]. Extracellular glycosylation typically involves enzymes secreted primarily by liver hepatocytes and platelets (glycosyltransferases). Changes in the composition of glycans have been documented in multiple inflammatory disorders and diverse forms of malignancies, often linked to the onset and progression of these pathological states [235,236].

The results indicate that an increase in the concentrations of fucosylated, sialylated, and agalactosylated glycans becomes apparent during the progression from steatosis to NASH and subsequent liver fibrosis [237]. Sialic acids fulfill a multitude of functions within glycolipids or glycoproteins, including the establishment of a protective barrier on cellular surfaces, the facilitation of interactions between leukocytes and the endothelial layer of blood vessels, as well as the recognition of pathogens, the binding of toxins, and the promotion of cell migration in certain oncological contexts [236]. Elevated concentrations of sialic acid in systemic circulation have demonstrated a significant correlation with metabolic syndrome and NAFLD [238]. Fucosylated glycans are implicated in a range of physiological and pathological mechanisms, including cellular adhesion, migration, angiogenesis, tumor dissemination, modulation of immune cells, and cellular proliferation [239]. In the liver, fucosylation acts as a signaling mechanism for the excretion of fucosylated glycoproteins from healthy hepatocytes into the bile [240]. However, in the presence of hepatocyte ballooning observed in NASH, the fucosylation-dependent sorting process may become dysfunctional, leading to the unintended release of fucosylated glycoproteins into the systemic circulation instead of the bile [239].

The majority of glycomic studies in NAFLD research have aimed to elucidate glycans or glycoproteins, which may function as potential blood biomarkers for differentiation between steatosis and NASH, as well as for the identification of liver fibrosis and its corresponding stage [46]. The diagnostic precision of most of these assays has been limited, with certain tests demonstrating enhanced accuracy in the diagnosis of NASH, while others excel in identifying advanced fibrosis [46]. These results suggest that, although alterations in the circulating glycome and glycoproteins are apparent in NAFLD, they are inadequate in isolation for the formulation of diagnostic models pertinent to the disease [46]. Thus, their integration with additional clinical or biochemical parameters is considered essential. Fucosylated-haptoglobin, for instance, exhibited an accuracy of below 70% in differentiating steatosis from NASH but reached 76–81% when combined with Mac2bp [241].

Similarly, in a study by Perakakis et al., which identified the largest number of glycans utilizing mass spectrometry to date, the serum concentrations of glycans were able to discriminate between the presence and absence of liver fibrosis with a sensitivity of 76% and specificity of 74% [215]. Although glycans alone exhibited a limited ability to differentiate between steatosis and NASH, their integration with lipid species in models encompassing 20 variables (comprising 18 lipid species and 2 glycans) significantly augmented both sensitivity and specificity in distinguishing between NASH, steatosis, and controls [46].

The suppression of fucosylation through the application of 2-fluorofucose has been shown to suppress proliferation, migration, and tumor formation in HepG2 liver cells [242]. It remains to be investigated whether analogous therapeutic strategies may yield advantageous outcomes in NAFLD by mitigating inflammatory processes, fibrosis processes, or the formation of HCC.

Figure 3 summarizes the metabolite markers and their application as potential markers for distinguishing the different stages of MASLD.

## 6. Exposomics in NAFLD

Exposomics is the examination of the exposome through various exposure assessment methodologies and is critical in liver pathology [243,244]. The term “exposome” was defined in 2005 and refers to all environmental exposures from conception, serving as a novel approach to identifying environmental disease risk factors [245]. The exposome encompasses a holistic health framework, incorporating social, chemical, radiotoxic, and physical elements [246].

Numerous factors affect the manifestation of NAFLD/MAFLD/MASLD, including dietary habits, lack of physical activity, disrupted circadian rhythms, substance abuse, and toxin exposure [243,247]. These factors influence pathological mechanisms such as metabolic processes, fibrosis, and inflammation [247]. Additionally, recent research highlights significant associations between environmental exposures and liver diseases, including air pollution and chemical contaminants, as well as radiation [243]. Environmental pollutants, such as metals and persistent organic pollutants (POPs), also influence such biological mechanisms. Per- and polyfluoroalkyl substances (PFAS), a notable subset of POPs, exhibit high persistence and accumulate in liver tissue. Their exposure correlates with negative health outcomes, including immunotoxicity and metabolic disorders like carcinogenesis [243]. Furthermore, these compounds impact the metabolism of carbohydrates, amino acids, bile, and lipids under certain conditions [248].

Additionally, microbial metabolites and the gut–liver axis significantly influence hepatic diseases [243]. More specifically, exposure to food contaminants correlates with dysbiosis and gut barrier impairment, resulting in modifications to the gut–liver axis and increased liver inflammation. Alterations in microbial metabolites further influence hepatic metabolic pathways, particularly lipid metabolism [243]. Various contaminants and pollutants stimulate multiple hepatic receptors, causing considerable metabolic disturbances [243]. The cumulative effects of these changes elevate the risk of liver-related diseases, including NAFLD, NASH, cirrhosis, and cancer. Exposomics is expected to significantly impact liver pathology research [243].

The liver’s pivotal function in metabolism involves interaction with other organs. An example is the “pancreas-liver” communication via glucagon and insulin, which regulate hepatic carbohydrate and lipid metabolism. The disruption of this interaction is implicated in various chronic liver diseases, such as NAFLD, and is influenced by the “gut-liver” axis, including gut microbiota that respond to dietary factors and contaminants [249].

Recently, it has been integrated with other omics, resulting in functional exposomics, which characterizes the biological implications of the exposome and its diverse exposures, analogous to functional genomics and the genome’s functional expression [244]. Advances in methodologies may elucidate the exposome’s influence on the liver, facilitating enhanced prevention, biomarker identification, and new therapeutic targets [243].

## 7. Discussion

The worldwide prevalence of NAFLD is on the rise, making it the leading cause of liver-related morbidity and mortality [10]. This global rise in NAFLD highlights the urgent need for reliable, non-invasive biomarkers to diagnose and stage steatosis, steatohepatitis, and fibrosis and to enable timely risk stratification and management [10]. Liver biopsy remains the gold standard for diagnosing NAFLD; however, its invasive nature and variability have prompted the exploration of non-invasive diagnostic alternatives [10]. Non-invasive diagnostic tools are becoming increasingly relevant, especially with advancements in omics technologies.

The emergence of high-throughput technologies has transformed biomedical research, further allowing multi-omic approaches for disease diagnosis, management, monitoring, treatment, and novel biomarker discovery [250]. The primary benefit of omics lies in generating extensive and unbiased data rapidly [46]. The recent advances in machine learning and artificial intelligence facilitate precise analysis of the large datasets produced by omics, resulting in a wealth of NAFLD-related insight [46]. These technologies present substantial opportunities to meet clinical demands, particularly in non-invasive NAFLD diagnosis and treatment. The key contributions of omics technologies in MASLD research are summarized in Table 1. Thus, initial proof-of-concept studies in this translational research domain represent valuable strides toward developing essential methodologies and tools for significant advancements in treating patients in the foreseeable future [46].

Of note, traditional approaches that focus on a single aspect of biology often miss the bigger picture, failing to capture the multifactorial nature of NAFLD. In contrast, multi-omics provide a more holistic view, helping to identify key regulatory pathways and biomarkers that are crucial to disease progression [251]. To decode intricate biological mechanisms, an integrative approach to analyzing multi-omics data is essential to elucidate the interconnections among biomolecules and their functional implications. Additionally, omics technologies present significant avenues for unraveling the underlying data associated with diseases [43].

**Figure 4:**
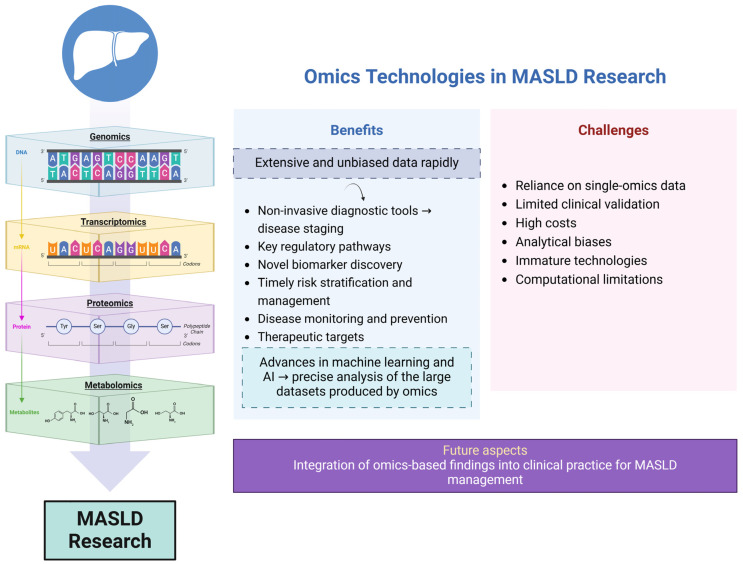
Omics technologies in MASLD research: benefits, challenges, and future directions. Created in BioRender. https://BioRender.com/n44h190 (accessed on 12 February 2025).

Despite the immense promise of omics technologies, several challenges remain (Figure 4). Translating omics-based biomarkers into clinical tests for MASLD has proven challenging due to disorganized research, reliance on single-omics data, and limited clinical validation [43,252]. High costs, analytical biases, and immature technologies further limit their clinical application [43]. Despite these challenges, advancements in omics platforms and decreasing costs are expected to make multi-omics approaches more accessible in clinical hepatology in the coming decade, potentially transforming the management of liver disease. Thus, significant advances are expected in the relevant evidence base and maturity of multi-omics, enabling the integration of omics-based biomarkers into clinical practice as precision tools for personalized medicine [43].

This shift toward integrating different data types has already led to significant breakthroughs. Thus, multi-omics offers a powerful tool for unraveling the complex molecular mechanisms behind NAFLD, paving the way for more targeted and personalized treatment approaches. This comprehensive framework holds great promise not only for enhancing disease prevention and treatment but also for improving long-term outcomes for patients [251].

Future research should investigate the landscape of MASLD, incorporating patient stratification in trials. Additionally, existing scores require validation under the newly introduced criteria, considering disease heterogeneity for comprehensive representation. Finally, investigating the cost-effectiveness of novel biomarkers and multi-omic approaches for disease monitoring is also crucial.

## 8. Concluding Remarks

Steatotic liver disease, including MASLD, ALD, and MetALD, affects a significant portion of the overall adult population and can lead to severe liver-related complications. Accurate risk stratification and effective treatments are urgently needed, as current biomarkers are insufficient for accurately diagnosing fibrosis and monitoring disease progression. Omics technologies—such as genomics, transcriptomics, proteomics, metabolomics, and exposomics—have advanced significantly, offering the potential for deeper insights into MASLD pathophysiology, diagnostics, and therapeutic targets.

These high-throughput technologies, which analyze various biological markers, are becoming more cost-effective and feasible for identifying disease markers across different sample types. Omics-driven approaches, combined with evolving computational tools, are improving the detection and prediction of MASLD. However, challenges remain, including concerns about disease specificity, costs, and computational limitations. Further validation through high-quality studies is essential before omics-based findings can be fully integrated into clinical practice for MASLD management. High-quality research and collaborative efforts will be pivotal in realizing this vision.

## Figures and Tables

**Table 1:** Key contributions of omics technologies in MASLD research.

Omics	Key Contributions of Omics in MASLD Research	References
Genomics	• Identification of genetic risk factors	[46,48,49,53]
	• Understanding disease mechanisms	[51]
	• Personalized treatment and risk stratification	[46,52]
	• Therapeutic target identification	[51]
	• Improved monitoring and management	[46]
	• Addressing population-specific challenges	[52]
Transcriptomics	• Insight into disease mechanisms	[106]
	• Gene expression regulation	[46,106]
	• Identification of disease pathways	[46,107,108,109,110,111,112]
	• Stratification of disease stages	[115]
	• Identification of biomarkers for diagnosis	[116,129,138]
Proteomics	• Identification of biomarkers and improved biomarker combinations	[169,179,180]
	• Non-invasive diagnostic tools	[119,171]
	• Disease stage differentiation	[169,173,185,186]
	• Uncover therapeutic targets	[168,172]
	• Personalized medicine potential	[168]
Metabolomics	• Comprehensive insight into disease mechanisms	[205]
	• Biomarker discovery—non-invasive diagnostic	[202,205]
	• Precision medicine applications (Algorithm development)	[215,216]
	• Real-time physiological status	[204,205]
	• Advancing therapeutics	[205]
Exposomics	• Holistic risk assessment	[243,247]
	• Insight into pathological mechanisms	[243,247,248]
	• Targeted prevention and intervention	[243,247,249]

## Data Availability

All data related to this research are available within the manuscript.
